# Shifts in Dissolved Organic Matter and Microbial Communities Under Continuous Cropping of *Aralia continentalis* Kitag.: A Comparative Study of 2-, 6-, and 12-Year Durations

**DOI:** 10.3390/biology14121750

**Published:** 2025-12-06

**Authors:** Qian Liu, Xingchi Guo, Ying Qu, Yuhe Xing, Junyan Zheng, Zhiyu Dong, Wei Yu, Guoyu Zhang

**Affiliations:** 1 College of Landscape Architecture, Changchun University, Changchun 130012, China; 2 Institute of Resource Utilization and Soil Conservation, Changchun University, Changchun 130022, China

**Keywords:** continuous cropping, *Aralia continentalis* Kitag., dissolved organic matter (DOM), 3D fluorescence spectroscopy, UV-visible spectroscopy, soil fertility, humification, microbial activity, soil health

## Abstract

Continuous cropping affects soil health, microbial diversity, and organic matter dynamics, but its long-term impacts on soils under *Aralia continentalis* Kitag. (a medicinally important Northeast China-native plant) remain unclear. This study evaluated effects of 2-, 6-, and 12-year continuous cropping on soil microbial communities, physicochemical properties, and dissolved organic matter (DOM) of bulk soils, and elucidated links between cropping duration and soil health indicators. Results showed that key physicochemical properties (total organic carbon, TOC) and available nutrients (available nitrogen, AN; available phosphorus, AP; available potassium, AK) declined with cropping duration: AN, AP, and AK decreased from 75.24 ± 1.2, 16.39 ± 0.05, and 104.8 ± 0.27 mg·kg^−1^ (2 years) to 63.47 ± 1.53, 13.38 ± 0.16, and 88.71 ± 0.94 mg·kg^−1^ (12 years), respectively. Microbial diversity increased initially but stabilized after 6 years, with communities shifting from copiotrophic taxa (e.g., Proteobacteria) to oligotrophic taxa (e.g., Acidobacteria). Partial Least Squares Path Modeling (PLS-PM) revealed strong positive correlations between dissolved/organic carbon (DOC/TOC) and microbial diversity, highlighting organic matter’s role in sustaining microbial richness. UV-visible and 3D fluorescence spectroscopy indices correlated significantly with microbial diversity, confirming their utility for monitoring DOM quality and microbial dynamics. This study clarifies dynamic interactions between soil properties, microbial diversity, and organic matter under continuous cropping, providing insights for sustainable cultivation of *A. continentalis*.

## Simple Summary

Continuous cropping can affect soil health, which is vital for growing crops sustainably. In this study, we looked at how growing *Aralia continentalis* Kitag. for 2, 6, and 12 years affects the soil around its roots. We found that over time, the soil health declined, particularly in terms of important nutrients and organic matter. Initially, the microbial diversity in the soil increased, but after 6 years, it stabilized. As the soil became poorer in nutrients, the types of microbes changed, with those needing fewer nutrients becoming more common. We also used UV-visible spectroscopy, 3D fluorescence spectroscopy, and Partial Least Squares Path Modeling (PLS-PM) to quantify the relationships between soil nutrients, microbes, and organic matter are related. We found that the more organic matter in the soil, the more diverse the microbes were. Additionally, UV-visible and 3D fluorescence spectroscopy showed that measuring the light absorbed by the soil could help us track the health of the soil and its microbes. These findings help us understand the long-term effects of continuous cropping and can guide farmers in using better practices to keep soil healthy, which is essential for sustainable farming.

## 1. Introduction

*Aralia continentalis* Kitag., a perennial herb belonging to the Araliaceae family (genus *Aralia*), is a medicinal plant native to Northeast China, renowned for its significant pharmacological properties, including anti-inflammatory, antioxidative, and anticancer effects [1]. Unlike plants from other families (e.g., Asteraceae with shallow root systems and high allelopathic compound secretion, or Fabaceae with root nodules for nitrogen fixation [2]), *A. continentalis* exhibits unique morphological and physiological traits: it develops a deep taproot system (extending 40–60 cm into the soil) that releases specific root exudates (e.g., saponins and polysaccharides [3]), which distinctly shape the composition of its rhizosphere microenvironment. This root-specific interaction with soil differentiates its rhizosphere/bulk soil dynamics from those of Asteraceae or Fabaceae crops, where microbial community assembly and organic matter decomposition are driven by shallow root exudation (Asteraceae) or symbiotic nitrogen fixation (Fabaceae) [4].

The focus on *A. continentalis*, rather than other crops, is justified by three key considerations: First, its medicinal value is economically and ecologically critical. The root of *A. continentalis* is a core ingredient in traditional Chinese medicines and modern pharmaceuticals targeting inflammatory diseases [5]. Due to overharvesting of wild populations, artificial cultivation—defined as large-scale planting of *A. continentalis* under standardized agricultural management practices (including deliberate site selection, seed sowing, organic-inorganic fertilization, drip irrigation, and pest control)—has become the primary source of this plant, with Northeast China contributing >70% of the national yield. Second, *A. continentalis* faces severe continuous cropping obstacles that are understudied. Unlike staple crops (e.g., maize, wheat) where continuous cropping impacts are mitigated by conventional fertilization, *A. continentalis* shows a 30–50% decline in root biomass and 20–30% reduction in saponin content after 5 years of continuous cropping, with the underlying mechanisms (e.g., root exudate-induced soil microbial imbalance) remaining unclear. Third, its cultivation in Northeast China’s black soil—an important soil type for Chinese agriculture—makes it a model system to study continuous cropping effects on soil health in temperate agroecosystems. Black soil is rich in organic matter but sensitive to cropping disturbances and understanding *A. continentalis*–soil interactions can inform sustainable management of other medicinal plants in similar soil regions [6].

The impact of monoculture cropping on soil properties is a well-documented issue. Continuous cultivation of a single crop can lead to the depletion of specific nutrients, alterations in soil structure, and shifts in microbial communities that affect soil health [7]. In monocultures, the lack of plant diversity often results in reduced soil organic matter, lower nutrient cycling efficiency, and the potential for increased soil-borne diseases. These soil property changes are often exacerbated by the repeated use of the same cropping system, leading to reduced long-term soil fertility and microbial imbalance. Monoculture crops such as *A. continentalis* may further exacerbate these issues, as they release specific root exudates that shape microbial communities, possibly creating a feedback loop that promotes soil degradation over time. Understanding the effects of monoculture cropping on soil properties, particularly in relation to soil organic matter and microbial dynamics, is essential for developing more sustainable agricultural practices and mitigating negative impacts on soil health.

However, its cultivation faces considerable challenges under continuous cropping systems, where soil degradation and microbial imbalances are common [8]. Continuous cropping can lead to soil nutrient depletion, alterations in soil structure, and shifts in the microbial communities that play a crucial role in plant health [9,10]. Understanding the effects of long-term cropping on soil properties, particularly in relation to soil organic matter and microbial communities, is essential for developing sustainable agricultural practices for this important medicinal plant [11].

Dissolved organic matter (DOM) in the soil is a key component in soil biogeochemical cycles, influencing nutrient availability and microbial activity [12]. DOM consists of a complex mixture of organic compounds released from plant roots and soil microbes, which play a pivotal role in soil structure, nutrient dynamics, and microbial community composition [13]. The composition and characteristics of soil DOM can significantly alter under different cropping durations, affecting soil fertility and the microbial ecosystem. However, it is important to acknowledge that soil microbial communities are influenced by a variety of factors beyond DOM, such as soil pH, moisture content, texture, and nutrient availability. These variables also contribute to shaping the microbial communities, but this study specifically focuses on the role of DOM due to its substantial impact on microbial dynamics and its direct involvement in nutrient cycling. Previous studies have highlighted the importance of DOM in shaping soil microbial communities, but the effects of continuous cropping on the soil DOM, particularly in medicinal plant systems, remain underexplored [14,15,16].

In addition to chemical and biological soil components, microbial diversity in the soil plays a vital role in maintaining soil health and plant growth [17]. Soil microbial communities, which consist of bacteria, fungi, and archaea, interact with plants and soil organic matter to drive nutrient cycling and disease suppression. The diversity and structure of these microbial communities are sensitive to soil management practices, including continuous cropping, which can lead to shifts in community composition and function [18]. However, the long-term impact of continuous cropping on microbial diversity, particularly in relation to medicinal plant cultivation, remains an area requiring further investigation [19].

This study aims to investigate the effects of continuous cropping on both the composition of soil DOM and microbial communities in the soil surrounding *Aralia continentalis* Kitag. The research specifically focuses on three cropping durations: 2 years (2 y), 6 years (6 y), and 12 years (12 y), to explore how these different durations influence soil properties, DOM characteristics, and alpha microbial diversity (richness and evenness) and bacterial community composition (phylum- and genus-level structural shifts). The study employs advanced analytical techniques, including 3D fluorescence spectroscopy and UV-visible absorption spectroscopy, to characterize the DOM, and bacterial community profiling to examine microbial diversity. By comparing these three cropping durations, we aim to provide insights into the long-term effects of continuous cropping on soil health and microbial dynamics, and further propose actionable agricultural management strategies (e.g., rational crop rotation, organic amendment addition, or microbial agent application) for sustainable cultivation of *Aralia continentalis* and similar medicinal crops [20,21].

Based on nutrient depletion and root exudates in continuous cropping systems, we propose the following hypotheses: i. If the duration of continuous cropping is extended, the availability of soil nutrients for *Aralia continentalis* will decrease, accompanied by a reduction in soil pH. ii. As the continuous cropping duration increases, the soil microbial community will shift, for instance, from microbes adapted to high-nutrient conditions toward those suited to low-nutrient environments. iii. Changes in microbial community richness will lead to corresponding alterations in soil dissolved organic matter (DOM). Specifically, higher microbial richness may enhance the stability of soil DOM. iv. Prolonged continuous cropping will alter soil dissolved organic matter (DOM), which in turn will drive changes in the soil microbial community.

## 2. Materials and Methods

### 2.1. Study Area and Soil Sampling

**Figure 1:**
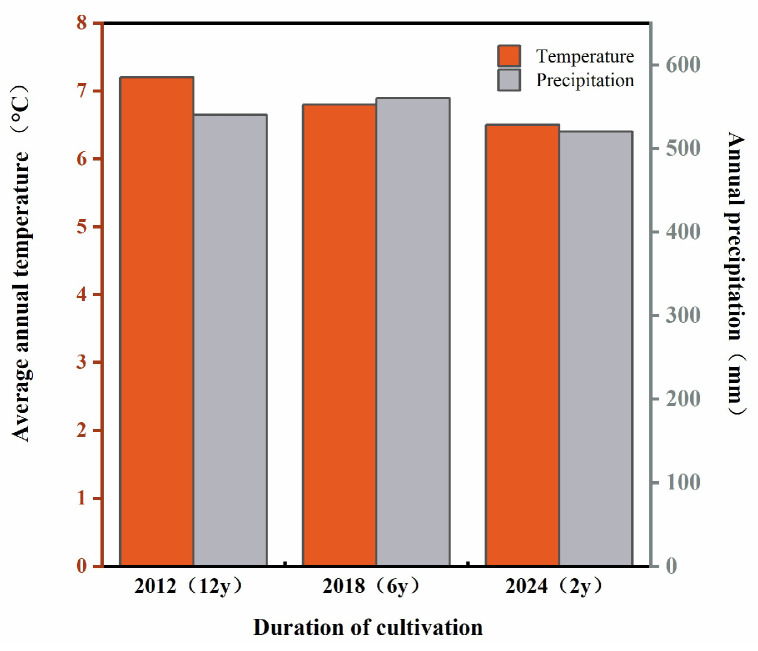
Annual average temperature and annual precipitation for different planting years.

The experiment was conducted at the experimental site of Changchun University, located in Changchun City, Jilin Province, China (43°50′9.4524″ N, 125°18′13.446″ E). The region is characterized by a temperate continental monsoon climate, with an average annual temperature of 6.9 °C (Figure 1). The area receives approximately 500–600 mm of precipitation annually, with the majority concentrated in the summer months, accounting for 60–70% of the total annual precipitation [22]. The soil at the site is classified as typical Northeast black soil, characterized by high organic matter content and good fertility. Its granulometric composition includes 40% sand, 30% silt, and 30% clay. Key physicochemical properties of the soil in 2012 were as follows: pH (H_2_O) 6.3, organic carbon content 22.5 g·kg^−1^, total nitrogen 1.2 g·kg^−1^, available phosphorus 15 mg·kg^−1^, and available potassium 110 mg·kg^−1^.

To establish experimental plots with different continuous cropping durations, *Aralia continentalis* Kitag. was planted in three separate batches at the same site: the first batch in autumn 2012 (to achieve 12 years of continuous cropping by 2024), the second batch in autumn 2018 (to achieve 6 years by 2024), and the third batch in autumn 2022 (to achieve 2 years by 2024). All batches followed identical local agricultural practices for fertilization (organic–inorganic mixed fertilizer: 20 t·ha^−1^ organic fertilizer + 300 kg·ha^−1^ compound fertilizer, N:P_2_O_5_:K_2_O = 15:15:15) and irrigation (drip irrigation, 200 mm·year^−1^) to eliminate confounding effects from management differences. These three cropping durations were defined as experimental groups: 2 y, 6 y, and 12 y. Each experimental plot measured 12 × 12 m, separated by 2 m buffer zones, with three 12 × 12 m subplots established per plant age group. Each subplot contained 25 plants of *Aralia continentalis* Kitag. per plot, with a planting density of 1.7 plants·m^−2^. In May 2024, a field survey and soil sampling were initiated. Based on the results of the field survey, bulk soil (soil outside the rhizosphere, 5–10 cm away from plant roots to avoid rhizosphere effects) from continuous cropping of *Aralia continentalis* Kitag. for 2 years (2 y), 6 years (6 y), and 12 years (12 y) were selected for this study.

At each sampling site, nine sampling points were arranged in an “S” shape pattern to ensure representative soil collection. Bulk soil was collected by carefully scraping the soil adhering to the roots, ensuring that only the soil closely associated with the roots was sampled. The soil from each sampling point was thoroughly mixed to create a composite sample. For transportation, each composite soil sample was individually placed into a sterile plastic bag (sealed tightly to avoid cross-contamination), and then each sealed plastic bag was transferred into a separate airtight container filled with dry ice. This double-sealing approach ensured the samples remained fresh and inhibited microbial activity during transit. In the laboratory, visible plant and animal residues, stones, and other impurities were removed. A portion of the samples was air-dried and passed through a 0.25 mm sieve for subsequent analysis of soil nutrients, water-soluble substances, and fluorescence components. The remaining samples were stored at −80 °C for soil bacterial community structure analysis.

### 2.2. Experimental Methods

#### 2.2.1. Soil Nutrient Determination

Soil water content (SWC) was determined by oven-drying the soil at 105 °C. Soil pH was measured by extracting the soil with a 1:5 soil-to-water ratio and using a pH meter. Total organic carbon (TOC) and dissolved organic carbon (DOC) were determined using the Shimadzu TOC-L Series analyzer [23,24]. Total nitrogen (TN) was measured using the Kjeldahl method. Total phosphorus (TP) was determined using the molybdenum–ammonium colorimetric method, and total potassium (TK) was measured by flame photometry [25,26,27]. Alkaline nitrogen (AN) content was determined using the alkaline diffusion method. Available phosphorus (AP) was measured using the acid molybdate–antimony colorimetric method, and available potassium (AK) was determined using ammonium acetate extraction [28].

#### 2.2.2. Bacterial Community Analysis

Microbial diversity was assessed using the Illumina NovaSeq sequencing platform with paired-end sequencing [29]. Bacterial sequences were primarily analyzed based on the 16S rRNA gene (V3 + V4 regions), while fungal sequences were analyzed based on the internal transcribed spacer (ITS) region, specifically ITS1_f [30]. Sequences and subsequent analysis were performed by Beijing Biomarker Technologies Co., Ltd., Beijing, China. For each microbial analysis, three biological replicates were performed. Each replicate was an independently sampled and processed sample to ensure the reliability and reproducibility of the results.

#### 2.2.3. Determination of Soil Water-Soluble Substances Using UV-Visible Absorption Spectroscopy

Each sample (2 g) was weighed and placed into a 50 mL centrifuge tube, followed by the addition of 30 mL distilled water. The sample was then placed in a SHA-2A refrigerated water bath shaker (Henglong, Jiangsu, china) and shaken at 60 °C for 1 h. After centrifugation, the soil suspension was filtered through quantitative filter paper into a 50 mL volumetric flask. An additional 20 mL of distilled water was added to the remaining sample without stirring, and it was shaken at 60 °C for 30 min. The sample was then centrifuged again, and the soil suspension was filtered into the volumetric flask and diluted to the 50 mL mark. The filtration was passed through a 0.22 μm micropore filter, and the filtered solution was divided into two 5 mL centrifuge tubes for UV-visible absorption spectroscopy and 3D fluorescence spectroscopy analysis.

The filtered soil suspension was collected to obtain the dissolved organic matter (DOM) extract. A batch of 5 mL centrifuge tube samples was taken for UV spectroscopy. Each sample of DOM extract was placed into a 10 mm quartz cuvette, and the UV-visible absorption spectrum was measured using UVmini-1280 spectrophotometer (Shimadzu Corporation, Kyoto, Japan). The scanning range was set from 200 to 700 nm, with a scan speed of 300 nm·min^−1^ and a wavelength interval of 1 nm. Absorbance at different wavelengths was used to determine the organic carbon content in the soil and the degree of humification, by analyzing the absorption peak position and intensity, which can help identify the types of organic compounds potentially present in the soil suspension.

All absorbance data were standardized based on DOC concentration to eliminate dilution effects. Prior to measurement, samples were filtered through a 0.45 μm membrane to remove particulate interference.

The selected UV parameters include SUVA_254_, SUVA_260_, SUVA_300_, E_2_/E_3_, E_4_/E_6_, and SR, which are used to characterize the aromaticity and molecular composition of dissolved organic matter (DOM). The parameters and their calculation formulas are as follows:

(1) $${\mathrm{SUVA}}_{x}=\frac{{\mathrm{UV}}_{x}\times 100}{{0.01\times C}_{\mathrm{DOC}}}$$

(2) $$E_{2}/E_{3}=\frac{UV_{254}}{UV_{365}}$$

(3) $$\mathrm{SR}=\frac{S_{275–295}}{S_{350–400}}$$

SUVA_254_: Reflects the content of aromatic C=C bonds and the abundance of hydrophobic organic components. Higher values indicate greater aromaticity and complex structural features.

SUVA_260_: Represents the degree of humification and molecular weight distribution, closely related to condensed aromatic ring structures.

SUVA_300_: Associated with high molecular weight humic substances and long-wavelength chromophores, reflecting the stability and redox activity of DOM [31].

E_2_/E_3_: Represents the ratio of absorbance at 254 nm and 365 nm, used to characterize the molecular weight of DOM. A higher ratio (>3.5) indicates low molecular weight DOM, while a lower ratio (<3.5) indicates high molecular weight DOM.

E_4_/E_6_: Represents the ratio of absorbance at 465 nm and 665 nm, used for humic substance classification. A ratio greater than 5 indicates fulvic acid (low humification), while a ratio less than 5 indicates humic acid (high humification) [32].

SR: The slope ratio (S_275–295_/S_350–400_) is used to indicate the source of DOM. A higher ratio (>1) suggests terrestrial input, while a lower ratio (<1) indicates aquatic or microbial sources.

S_300~700_: Represents the UV slope in the 300–700 nm range, providing a comprehensive evaluation of DOM characteristics. A higher slope may indicate photodegradation or active microbial metabolism of DOM.

S_275~295_: Represents the UV slope in the 275–295 nm range, which reflects the molecular weight of DOM. A higher slope indicates smaller DOM molecules.

S_350~400_: Represents the UV slope in the 350–400 nm range, used to further analyze DOM composition. Combined with S_275~295_, this helps distinguish the sources of DOM [33].

#### 2.2.4. Determination of Soil Water-Soluble Substances Using 3D Fluorescence Spectroscopy—Peak Search Method

A batch of 5 mL centrifuge tube samples was taken, and the DOM extract from the soil suspension was placed in a 10 mm four-way optical quartz fluorescence cuvette. The samples were analyzed using the F-7000FL fluorescence spectrophotometer (Hitachi High-Tech Corporation, Tokyo, Japan), and three-dimensional fluorescence spectra (3D-EEMs) were obtained. It should be noted that the soil suspension was obtained through sequential hot-water extraction at 60 °C, followed by centrifugation and filtration through 0.22 μm membranes to isolate the water-soluble fraction of soil organic matter (DOM). The UV-Vis absorbance spectra were also recorded from 200 to 700 nm, providing information on the quality of DOM, including parameters such as SUVA_254_, SUVA_254_, SUVA_300_, E_2_/E_3_, E_4_/E_6_, and SR [34,35,36]. The excitation wavelength (Ex) scanning range was set from 200 to 500 nm, and the emission wavelength (Em) scanning range was set from 220 to 600 nm. Both the Ex and Em wavelength scanning intervals were set to 5 nm, with the excitation and emission monochromator slit widths set at 5 nm, and the scan speed was 1200 nm·min^−1^. This method is used to identify fluorescent substances, such as humic-like and protein-like substances, in the soil suspension. Humic-like substances exhibit fluorescence peaks at relatively fixed excitation and emission wavelengths, and protein-like substances also have characteristic fluorescence peak positions, allowing for the distinction of different substances. Organic materials of endogenous (internal) origin differ from those of exogenous (external) input based on their fluorescence spectra. As degradation progresses, the intensity and position of the fluorescence peaks will change. The fluorescence intensity of humic substances may decrease during degradation, and the peak position may shift either to the red (red shift) or blue (blue shift) end of the spectrum, reflecting the degradation process.

The fluorescence peaks, denoted as B, T, A, M, and C, represent the intensity of different types of DOM: B (Tyrosine-like); T (Tryptophan-like); A (Excitation humic-like); M (Marine humic-like); C (Terrestrial humic-like) [37].

Higher peak intensities indicate a greater concentration of the corresponding substance. Peaks B and T are associated with protein-like materials, representing fresher organic matter or biologically more active organic matter. Peaks A, M, and C are related to humic-like substances, which are more stable and complex organic materials, typically derived from the degradation of plant residues or long-term humification processes [38].

(4) $$\mathrm{FI}=\frac{I_{450}}{I_{500}}$$

(5) $$\mathrm{HIX}=\frac{\Sigma_{435\sim 480}I}{\Sigma_{300\sim 345}I}$$

(6) $$\mathrm{BIX}=\frac{I_{380}}{I_{430}}$$

FI (Fluorescence Index): FI is the ratio of fluorescence intensity at Ex 370 nm/Em 450 nm to Em 500 nm, used to distinguish DOM sources. An FI > 1.9 indicates a dominant autochthonous (self-generated) source, while an FI < 1.4 indicates a dominant allochthonous (external) source.

HIX (Humification Index): HIX is the ratio of the integral fluorescence intensity at Ex 254 nm/Em 435–480 nm to that at 300–345 nm, used to assess the degree of humification. An HIX > 6 indicates high humification, while an HIX between 4 and 6 indicates highly humified material with slight autochthonous characteristics. HIX < 4 indicates low humification.

BIX (Biological Index): BIX is the ratio of the fluorescence intensity at Ex 310 nm/Em 380 nm to Em 430 nm, used to evaluate the contribution of autochthonous sources. BIX > 1 indicates a strong autochthonous source, while BIX < 0.7 indicates a terrestrial-dominated source [39].

### 2.3. Statistical Analysis

Statistical significance was analyzed using the Least Significant Difference (LSD) method in IBM SPSS Statistics 26 (*p* < 0.05). Alpha diversity indices (e.g., Shannon index, Chao1 index, and observed species) were calculated to assess microbial diversity using QIIME2 (version 2021.8) for both bacterial and fungal communities. Three-dimensional fluorescence spectra and the delineation of fluorescence regions were created using Origin 2024 software. Peak concentration calculations for the 3D fluorescence spectra were performed using the peak search method in the RStudio 4.4.2 package. Partial Least Squares Path Modeling (PLS-PM) to assess the impact pathways of soil properties was conducted using R language (version 4.4.2).

## 3. Results

### 3.1. Changes in Soil Physicochemical Properties Due to Continuous Cropping

#### 3.1.1. Soil pH

**Figure 2:**
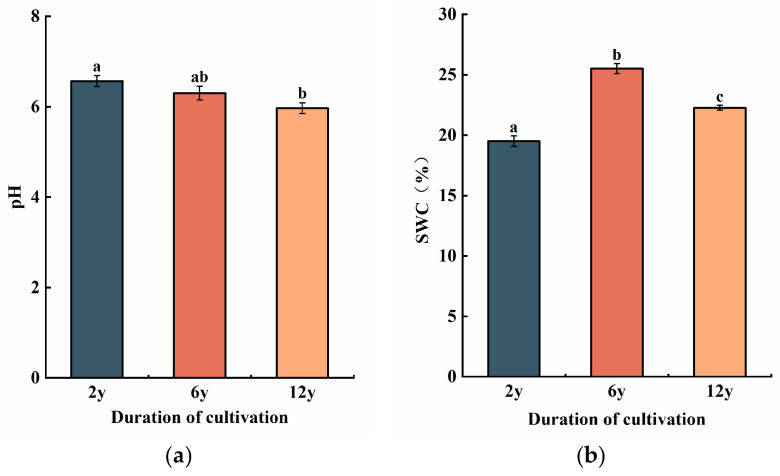
(**a**) Soil pH across different cultivation durations (2 y, 6 y, and 12 y). The vertical axis denotes soil pH, an indicator of soil acidity or alkalinity. (**b**) Soil water content (SWC, %) across different cultivation durations (2 y, 6 y, and 12 y). The vertical axis denotes SWC, expressed as a percentage. Different lowercase letters above the bars indicate significant differences among treatments at *p* < 0.05.

The soil pH showed a significant decrease from 2 y to 6 y and 12 y, with the lowest pH found in the 12-year continuous cropping soil. Specifically, the pH of the 2 y soil was higher (6.2 ± 0.1), while the 12 y soil showed a noticeable drop (5.7 ± 0.2) (Figure 2a).

#### 3.1.2. Soil Water Content (SWC)

Soil water content varied significantly across the three cropping durations, with the highest value observed in the 6 y soil (Figure 2b). The SWC in the 2 y soil was 19.6 ± 1.2%, while the 6 y soil exhibited the highest SWC at 22.3 ± 1.5%, and the 12 y soil had a lower value of 18.2 ± 1.3%.

**Figure 3:**
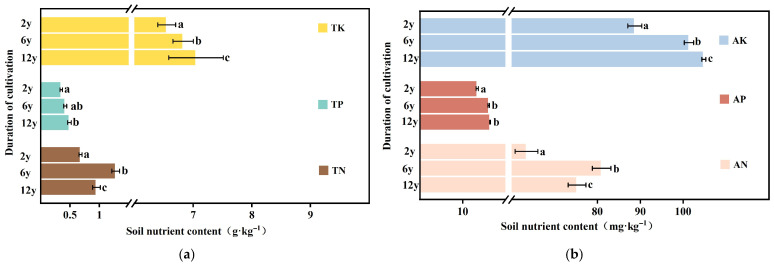
Soil nitrogen, phosphorus, and potassium contents under different continuous cropping durations. (**a**) Contents of total nitrogen (TN), total phosphorus (TP), and total potassium (TK). The vertical axis denotes the duration of cultivation (2 y, 6 y, 12 y), and the horizontal axis represents soil nutrient content with units of g·kg^−1^. (**b**) Contents of available nitrogen (AN), available phosphorus (AP), and available potassium (AK). The horizontal axis denotes soil nutrient content with units of mg·kg^−1^. Different lowercase letters above the bars indicate significant differences between groups at *p* < 0.05.

The results presented in Figure 3a clearly show that the concentrations of total nitrogen (TN), total phosphorus (TP), and total potassium (TK) in the soil were significantly influenced by the duration of continuous cropping. The contents of available nitrogen (AN), available phosphorus (AP), and available potassium (AK) also exhibited noticeable changes over the 2-year, 6-year, and 12-year cropping durations. A significant decrease in TN content was observed as the cropping duration increased. The 2-year continuous cropping soil had the highest TN content (0.95 ± 0.04 g·kg^−1^), while the 12-year soil showed the lowest TN content (0.68 ± 0.01 g·kg^−1^). The 2-year soil had the highest TP content (0.49 ± 0.02 g·kg^−1^), while the 12-year soil exhibited the lowest value (0.35 ± 0.01 g·kg^−1^). Total potassium content showed a slight decline as cropping duration increased, from 9.0 ± 0.27 g·kg^−1^ in the 2-year soil to 6.55 ± 0.09 g·kg^−1^ in the 12-year soil.

Available nitrogen (AN), available phosphorus (AP), and available potassium (AK) the key indices of soil nutrient availability—exhibited a significant decreasing trend with increasing cropping duration (Figure 3b). The 2-year soil had the highest AN content (75.24 ± 1.2 mg·kg^−1^), while the 12-year soil exhibited a much lower AN value (63.47 ± 1.53 mg·kg^−1^). Available phosphorus content followed a similar pattern, with the 2-year soil exhibiting the highest value (16.39 ± 0.05 mg·kg^−1^) and the 12-year soil the lowest (13.38 ± 0.16 mg·kg^−1^). Available potassium content showed a marked decrease from the 2-year soil (104.8 ± 0.27 mg·kg^−1^) to the 12-year soil (88.71 ± 0.94 mg·kg^−1^).

**Figure 4:**
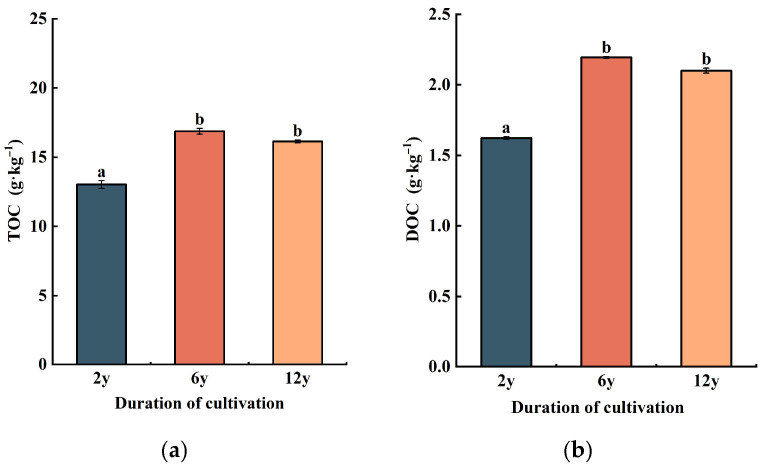
Soil organic carbon contents under different cultivation durations. (**a**) Soil total organic carbon (TOC) content. The vertical axis denotes TOC, with the unit of g·kg^−1^, representing the total amount of organic carbon in soil. (**b**) Soil dissolved organic carbon (DOC) content. The vertical axis denotes DOC, with the unit of g·kg^−1^, referring to the dissolved fraction of organic carbon in soil that is readily available for biological utilization. The horizontal axis the duration of cultivation 2 years, 6 years, and 12 years of cultivation, respectively. Different lowercase letters above the bars indicate significant differences between groups at *p* < 0.05.

The total organic carbon (TOC) content was highest in the 6-year continuous cropping soil (16.3 ± 0.9 g·kg^−1^), followed by the 12-year soil (14.2 ± 1.0 g·kg^−1^), and the lowest in the 2-year soil (12.4 ± 0.7 g·kg^−1^) (Figure 4a).

The dissolved organic carbon (DOC) concentrations were highest in the 6-year cropping soil (2.1 ± 0.2 g·kg^−1^), followed by the 12-year cropping soil (1.9 ± 0.1 g·kg^−1^), and the lowest in the 2-year cropping soil (1.3 ± 0.1 g·kg^−1^) (Figure 4b).

### 3.2. UV-Visible and Fluorescence Characteristics of DOM in Soil Leachate

#### 3.2.1. UV-Visible Spectral Characteristics of Soil DOM

**Figure 5:**
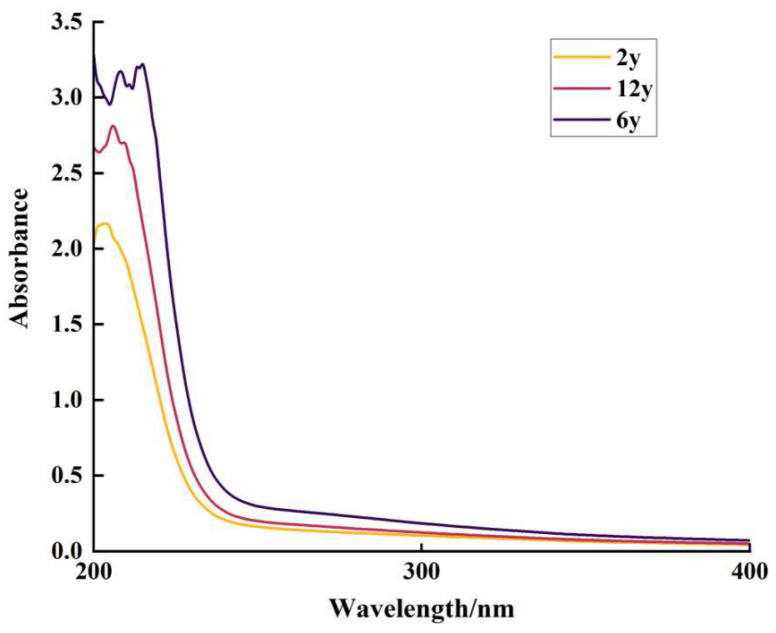
UV-visible absorption spectra of soil suspension at different continuous cropping durations. The horizontal axis denotes wavelength (nm), ranging from 200 nm to 400 nm, representing the range of ultraviolet-visible light. The vertical axis denotes absorbance, a dimensionless quantity that reflects the degree of light absorption by the soil suspension. Different colored curves correspond to soil suspension from continuous cropping durations of 2 years (2 y), 6 years (6 y), and 12 years (12 y), respectively.

The UV-visible absorption spectra of dissolved organic matter (DOM) in the soil leachate under different consecutive cropping durations (2 y, 6 y, and 12 y) were analyzed. As shown in Figure 5, the absorbance spectra exhibited clear differences among the three cropping durations. The absorbance in the UV region (200–400 nm) was significantly higher in the 2-year (2 y) soil compared to the 6-year (6 y) and 12-year (12 y) soils. Specifically, the absorbance peaks in the 2 y soil were more prominent, with the maximum absorbance reaching over 3.0 at approximately 250 nm, while the 12 y soil had lower absorbance values, peaking at around 2.5.

#### 3.2.2. Fluorescence Characteristics of Soil DOM

The fluorescence characteristics of dissolved organic matter (DOM) in the soil leachate were analyzed across the three consecutive cropping durations (2 y, 6 y, and 12 y). The data, presented in Table 1, show significant changes in various UV fluorescence parameters, which reflect the evolution of the molecular composition and aromaticity of the DOM.

As observed, SUVA_254_ values decreased from 2-year (2 y) to 12-year (12 y) continuous cropping of *Aralia continentalis* Kitag. The 2 y soil had the highest SUVA_254_ value (0.8679 ± 0.00816 L·mg^−1^·m^−1^), while the 12 y soil showed a significant decrease in SUVA_254_ value (0.8337 ± 0.0126 L·mg^−1^·m^−1^). SUVA_254_ and SUVA_300_ exhibited the same trend, with significantly higher values in the 2 y soil than in the 12 y soil.

In addition to the SUVA parameters, the E_2_/E_3_ and E_4_/E_6_ ratios were also significantly affected by continuous cropping. The E_2_/E_3_ ratio was highest in the 2 y soil (3.2816 ± 0.0046), while the E4/E6 ratio showed the highest value in the 2 y soil (17.86 ± 0.03043). As the cropping duration increased, both the E_2_/E_3_ and E_4_/E_6_ ratios declined.

The SR ratio, which reflects the source of dissolved organic matter (DOM), showed an increasing trend from the 2-year (0.85 ± 0.00527) to the 12-year (0.931 ± 0.0079) continuous cropping soils.

### 3.3. Fluorescence Components and Spectral Parameters from Soil 3D-EEM Spectra Under Continuous Cropping

#### 3.3.1. Changes in Fluorescence Components

**Figure 6:**
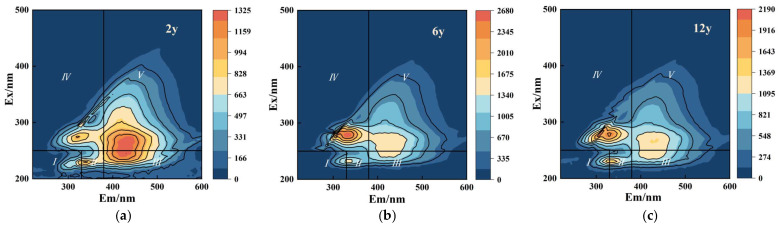
Three-dimensional fluorescence excitation-emission matrix (EEM) spectra of soil dissolved organic matter (DOM) under different consecutive cropping years (2 y, 6 y, and 12 y, corresponding to (**a**), (**b**), and (**c**), respectively). The horizontal axis (Em/nm) denotes emission wavelength, and the vertical axis (Ex/nm) denotes excitation wavelength. The color bar represents fluorescence intensity. The color bar represents fluorescence intensityThe 3D-EEM spectra are divided into five regions based on the regional integration method: Region I (Ex < 250 nm, Em < 330 nm) and Region II (Ex < 250 nm, 330 nm < Em < 380 nm) represent protein-like substances (e.g., tyrosine and tryptophan derivatives); Region III (250 nm < Ex < 280 nm, Em < 380 nm) represents soluble microbial by-product-like substances; Region IV (280 nm < Ex < 350 nm, Em < 480 nm) and Region V (Ex > 350 nm, Em > 480 nm) represent humic-like substances (e.g., fulvic acid and humic acid derivatives). These regions reflect the compositional characteristics of DOM under different consecutive cropping durations.

The 3D fluorescence spectra of dissolved organic matter (DOM) in the soil leachate under different continuous cropping durations (2 y, 6 y, and 12 y) showed distinct changes in the fluorescence components associated with organic matter degradation and humification processes (Figure 6a–c, Table 2), with corresponding alterations in fluorescence peak intensities and the distribution of fluorescence regions. In the 2 y cropping soil, the fluorescence spectra exhibited intense peaks in regions I and II (Ex/Em 280/320 nm and Ex/Em 300/420 nm). With increasing cropping duration, significant shifts were observed in the fluorescence spectra: in the 6 y soil, the fluorescence intensity in regions I and II decreased, while the peaks in regions IV and V (Ex/Em 400/480 nm and Ex/Em 420/500 nm) became more prominent; in the 12 y soil, the fluorescence intensity in regions I and II continued to decline, and the peaks in regions IV and V became more pronounced. Overall, continuous cropping significantly altered the composition of DOM in the rhizosphere, which shifted from protein-like to humic-like substances over time.

#### 3.3.2. Alterations in Spectral Parameter Characteristics

The fluorescence indices (FI), humification index (HIX), and biological index (BIX) of dissolved organic matter (DOM) in the soil leachate were evaluated under different continuous cropping durations (2 y, 6 y, and 12 y). As shown in Table 3, the FI values were highest in the 2 y cropping soil (1.7428 ± 0.01012), followed by the 6 y soil (1.7438 ± 0.0163), and the lowest in the 12 y soil (1.6875 ± 0.00885). The HIX, which reflects the degree of humification of DOM, showed a decrease with increasing cropping duration. The 2 y soil had the highest HIX value (0.7307 ± 0.00668), while the 12 y soil had the lowest HIX value (0.6396 ± 0.00689). Similarly, the BIX, which is used to estimate the biological source of DOM, exhibited a slight decrease over time. The 2 y soil had the highest BIX value (0.6811 ± 0.00502), followed by the 6 y soil (0.7358 ± 0.00809), and the lowest value was observed in the 12 y soil (0.6954 ± 0.00739).

#### 3.3.3. Distribution of Fluorescent Components in Soil DOM

**Figure 7:**
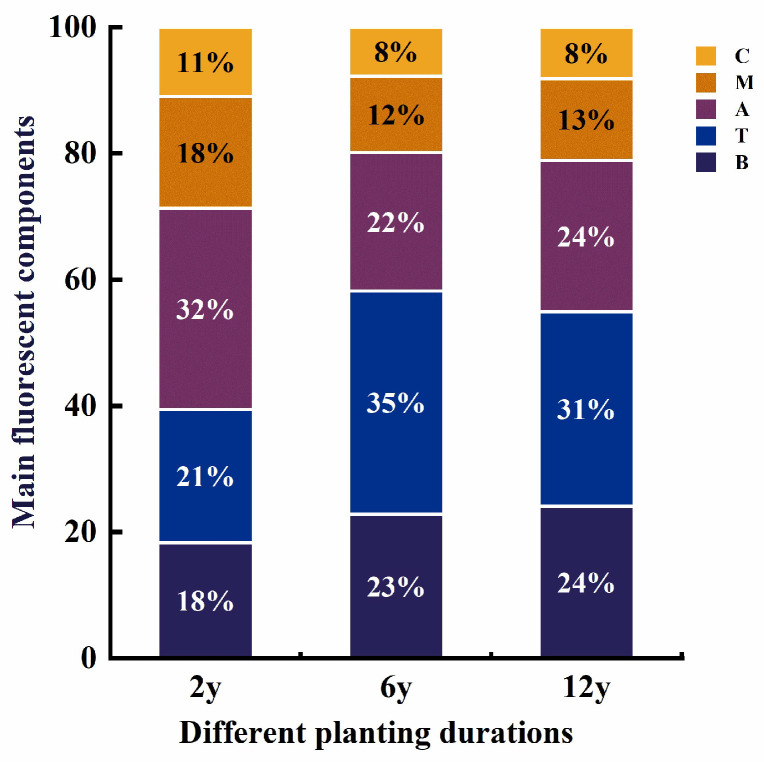
Proportions of main fluorescent components in soil dissolved organic matter (DOM) under different planting durations (2 y, 6 y, 12 y) identified by the three-dimensional fluorescence peak-finding method. The horizontal axis denotes different planting durations, and the vertical axis represents the proportion of each fluorescent component (%). The legends C, M, A, T, and B represent different fluorescent components: C (humic-like substance), M (microbial humic-like substance), A (fulvic acid-like substance), T (tryptophan-like substance), and B (tyrosine-like substance), respectively.

The distribution of the main fluorescent components in the dissolved organic matter (DOM) of the soil leachate was evaluated under different continuous cropping durations (2 y, 6 y, and 12 y). As shown in Figure 7, the relative contributions of protein-like (C), humic-like (M), terrestrial humic-like (T), and biological humic-like (B) substances to the overall fluorescence spectra were calculated for each cropping duration. The results indicate a distinct shift in the composition of fluorescent components with the increase in cropping duration. In the 2 y cropping soil, protein-like components (C) contributed the highest proportion to the overall fluorescence (32%), followed by humic-like components (M, 21%) and terrestrial humic-like substances (T, 18%). In the 6 y cropping soil, the relative contribution of protein-like components (C) decreased to 18%, while humic-like substances (M) increased to 32%, and terrestrial humic-like components (T) rose to 21%. In the 12 y cropping soil, the proportion of protein-like components (C) further decreased to 11%, while humic-like substances (M) remained significant (32%) and terrestrial humic-like substances (T) reached 24%.

### 3.4. Soil Microbial Bacterial Diversity and Composition

#### 3.4.1. Bacterial Community Composition (Key Facet of Beta Diversity)

**Figure 8:**
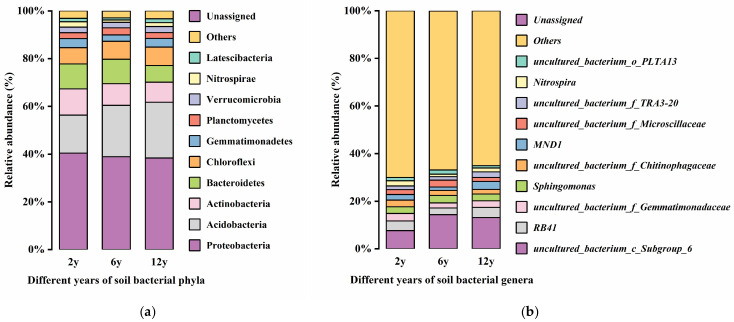
Changes in bacterial community structure: (**a**) Changes in bacterial community structure at phylum level, (**b**) changes in bacterial community structure at genus level.

The structure of the soil bacterial community was significantly influenced by continuous cropping over different time scales (Figure 8). At the phylum level (Figure 8a), Proteobacteria dominated the early stages of cropping (2 years) with a relative abundance of about 40%, which gradually decreased to 38% at 12 years. Conversely, Acidobacteria increased in relative abundance from 15% at 2 years to 22% at 6 years and remained stable at this level. Other bacterial phyla such as Actinobacteria and Bacteroidetes showed a declining trend over time, from 10% to 7%. Notably, Chloroflexi increased from 10% to 13% at 12 years. At the genus level (Figure 8b), *Nitrospira* showed a decline in relative abundance over time, particularly at 12 years. Additionally, *Sphingomonas* and MND1 were more abundant in the 12-year samples. The increase in Gemmatimonadetes at 12 years was also observed.

#### 3.4.2. Changes in Bacterial Community Diversity

Soil microbial alpha diversity (richness and evenness) was assessed using several indices, including OTU (operational taxonomic unit, reflecting richness), ACE (richness estimator), Chao1 (richness estimator), Simpson (evenness index), and Shannon (diversity index integrating richness and evenness) (Table 4). The results showed that the 2-year group had the highest OTU (1673.33 ± 5.51), ACE (1683.44 ± 5.16), and Chao1 (1686.90 ± 4.77) values. The 6-year and 12-year groups showed relatively stable OTU values (1693.67 ± 4.51 and 1696.00 ± 4.58, respectively). The Simpson index remained consistent at approximately 0.004 across all groups, while the Shannon index decreased slightly from 6.376 ± 0.017 at 2 years to 6.327 ± 0.017 at 6 years, before returning to 6.376 ± 0.014 at 12 years. Statistical analysis revealed significant differences in OTU, ACE, and Chao1 indices between the 2-year and 6-year groups, but no significant differences were observed between the 6-year and 12-year groups.

### 3.5. Correlation Between Soil Nutrients, Microbial Communities, and Spectral Indices

**Figure 9:**
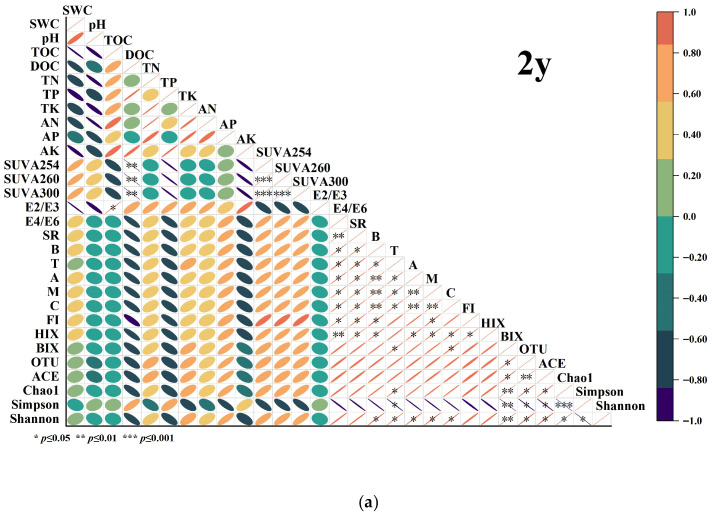

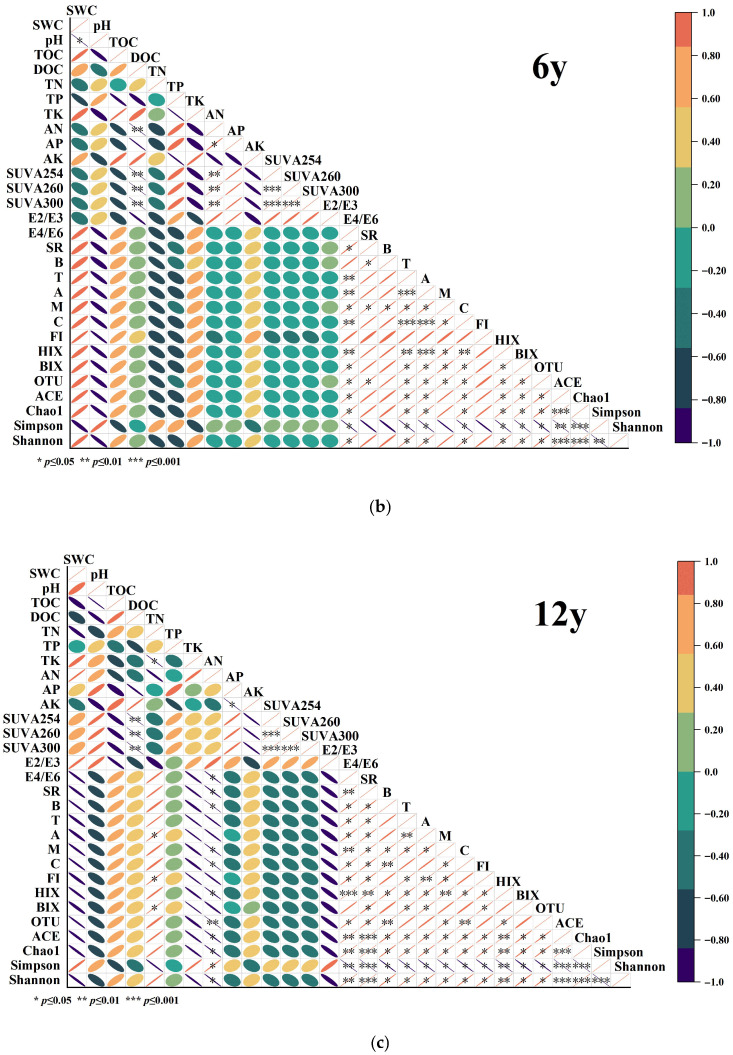
Correlation matrix of soil nutrients, bacterial diversity indices, UV absorption indices, and 3D fluorescence spectroscopy indices at 2 years (**a**), 6 years (**b**), and 12 years (**c**) of continuous cropping. *, **, and *** indicate significant (*p* < 0.05), highly significant (*p* < 0.01), and extremely significant (*p* < 0.001) correlations, respectively. The orientation of the ellipse indicates the direction of the correlation. A rightward tilt indicates a positive linear relationship between variables. A leftward tilt indicates an inverse or negative linear relationship. SWC (Soil Water Content), pH (soil acidity–alkalinity), TOC (Total Organic Carbon), DOC (Dissolved Organic Carbon), TN (Total Nitrogen), TP (Total Phosphorus), TK (Total Potassium), AN (Available Nitrogen), AP (Available Phosphorus), AK (Available Potassium); SUVA_254_, SUVA_260_, SUVA_300_ (Specific UV Absorbance at 254 nm, 260 nm, 300 nm, indicating DOM aromaticity and molecular weight), E_2_/E_3_ (Absorbance ratio at 250 nm/365 nm), E4/E6 (Absorbance ratio at 465 nm/665 nm), SR (Spectral Ratio, reflecting DOM humification and molecular weight); (Tyrosine-like substance), T (Tryptophan-like substance), A (Fulvic acid-like substance), M (Microbial humic-like substance), C (Humic acid-like substance); FI (Fluorescence Intensity, total content of fluorescent substances), HIX (Humification Index, DOM humification degree), BIX (Biological Index, biological source contribution to DOM); OTU (Operational Taxonomic Unit), ACE, Chao1 (richness estimators), Simpson, Shannon (diversity indices).

Correlation analysis revealed distinct temporal dynamics in the relationships between soil physicochemical properties and soil biological characteristics (predominantly reflected by microbial diversity indices) across the continuous cropping chronosequence. In the 2-year soil samples (Figure 9a), soil organic carbon (TOC) exhibited a significant positive correlation with the Shannon diversity index. Dissolved organic carbon (DOC) showed positive correlations with both the Simpson and ACE indices. In contrast, SUVA_254_ was negatively correlated with both ACE and Chao1 indices. In the 6-year soil samples (Figure 9b), a significant negative correlation emerged between soil pH and both the Simpson and Shannon indices. DOC maintained positive correlations with the ACE index and OTU. In the 12-year soil samples (Figure 9c), the negative correlation of SUVA_254_ strengthened, showing significant negative relationships with both the Shannon and Simpson indices. DOC continued to demonstrate positive correlations with these same diversity indices.

### 3.6. Correlation Between Soil Nutrients, Microbial Diversity, and Spectral Indices

**Figure 10:**
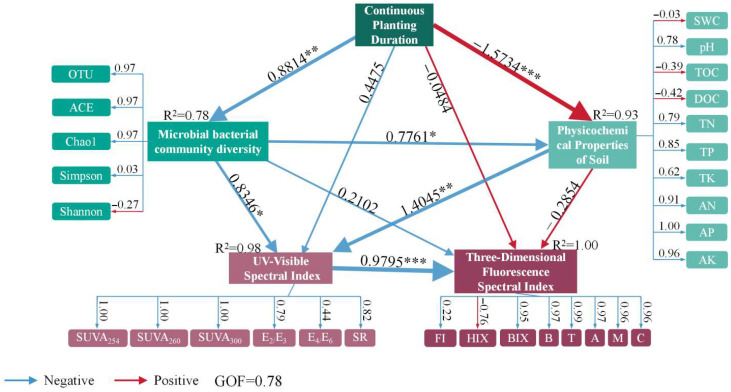
Path Analysis of Soil Nutrients, Bacterial Diversity, and Spectral Indices in Continuous Cropping Systems. This Partial Least Squares Path Modeling (PLS-PM) diagram illustrates the causal relationships among drivers (continuous cropping duration, soil physicochemical properties, bacterial community diversity) and spectral proxies (UV-visible and 3D fluorescence indices) under continuous cropping. Spectral indices are optical indicators of soil organic matter (SOM) quality (aromaticity, humification) and microbial dynamics, while drivers directly/indirectly regulate SOM transformation and spectral characteristics. The model’s Goodness of Fit (GOF = 0.78) and high R^2^ values (UV-visible: 0.78; 3D fluorescence: 0.98) reflect strong ecological coupling between drivers and spectral responses, consistent with soil biogeochemical processes. *, **, *** indicate significant correlations at *p* < 0.05, *p* < 0.01, *p* < 0.001, respectively.

The Partial Least Squares Path Modeling (PLS-PM) analysis (Figure 10) effectively quantified the complex relationships within the continuous cropping soil system, demonstrating a high overall explanatory power with a Goodness of Fit (GOF) index of 0.78. The model explained 78% of the variance in UV-visible spectral indices, 98% in 3D fluorescence spectral indices, and 93% in soil physicochemical properties (Figure 9). A significant negative correlation was identified between continuous cropping duration and soil physicochemical properties (R^2^ = 0.93, *p* < 0.001). In contrast, a positive correlation was observed between cropping duration and microbial community diversity indices (R^2^ = 0.78, *p* < 0.001). Furthermore, these microbial diversity indices (e.g., OTU, ACE, Shannon) showed significant positive correlations with both UV-visible spectral indices (R^2^ = 0.98, *p* < 0.001) and 3D fluorescence spectral indices (R^2^ = 0.9795, *p* < 0.001). Specific indices such as the Shannon and Simpson indices exhibited strong positive correlations with dissolved organic carbon (DOC) and total organic carbon (TOC).

## 4. Discussion

### 4.1. Effects of Continuous Cropping on Soil Physicochemical Properties

This decline in pH (Figure 2a) could be attributed to the accumulation of organic acids from continuous organic matter decomposition and reduced microbial diversity over extended periods of monocropping [40,41]. The lower pH in long-term continuous cropping may also be linked to nutrient leaching and soil acidification, which often occur under intensive agricultural practices [42,43].

It is important to note that variations in soil water content (SWC) can be influenced by several factors, including recent weather conditions, irrigation practices, and sampling time. Therefore, while the observed differences across cropping durations may suggest a trend, it is not conclusive to attribute the variations solely to cropping history without controlling for these external factors. Further studies that control environmental variables, such as precipitation and irrigation, are necessary to definitively assess the impact of cropping history on soil water retention. The decrease in SWC (Figure 2b) in the 12 y soil could be due to a combination of reduced soil structure stability, lower organic matter input, and microbial degradation, which may impair the soil’s ability to retain moisture efficiently [44,45].

The observed decline in total nitrogen (TN) likely reflects depleted nitrogen reserves, reduced nitrogen fixation, and diminished soil microbial activity (critical for nitrogen cycling) under prolonged cropping [46,47]. This is consistent with continuous cropping-induced exhaustion of available nitrogen pools—unreplenished in monocultures lacking crop rotation or organic amendments. Reduced microbial diversity/activity further worsens nitrogen depletion by impairing nitrogen mineralization and organic nitrogen turnover, leading to net soil nitrogen loss and reduced fertility over time [48,49]. Available potassium (AK) also showed gradual depletion (though less pronounced than TN/TP), driven by ongoing plant uptake and leaching—potassium is highly mobile in soil, especially under intensive agriculture, and its depletion harms plant health [50].

The reduction in core nutrients (nitrogen, phosphorus, potassium) is linked to altered nutrient cycling dynamics: long-term continuous cropping modifies microbial composition, which in turn affects nitrogen mineralization, phosphorus availability, and potassium dynamics [51]. The absence of organic amendments and crop rotation compromises the soil’s ability to replenish these nutrients, while leaching (particularly of potassium and phosphorus) accelerates depletion [52]. Collectively, these data confirm that key nutrient indicators (TN, TP, AK) and total nutrient contents (TN, TP, TK) decline with prolonged continuous cropping, highlighting the need for nutrient management in monocultures [53].

For organic carbon: In the early cropping stage (2 y), *Aralia continentalis*’ juvenile growth (underdeveloped roots, limited biomass) leads to low plant residue input and thus low total organic carbon (TOC). By 6 y, mature plants (2.3-fold higher root biomass, increased litterfall) drive greater organic carbon input than microbial decomposition, promoting TOC accumulation. By 12 y, slight plant growth decline (due to long-term nutrient limitation) still results in higher cumulative residue input than 2 y soils, though TOC decreases—possibly from depleted organic inputs and altered microbial activity impairing carbon retention [54].

Dissolved organic carbon (DOC) was highest under 6 y continuous cropping, likely from enhanced microbial decomposition of organic matter releasing more soluble carbon. In contrast, 2 y soils had lower DOC (limited easily decomposable organic matter in early plant growth), while 12 y soils showed reduced DOC (microbial dynamics limiting soluble carbon mobilization over time) [55,56,57].

### 4.2. The Effect of Dissolved Organic Matter (DOM) in Soil on the Changes in Ultraviolet Parameters

#### 4.2.1. Influence of UV-Visible Spectral Properties of Soil DOM

The more prominent absorbance peak in the 2-year (2 y) soil indicates a higher concentration of aromatic organic compounds in its dissolved organic matter (DOM)—consistent with the strong UV-absorption properties of such compounds. Across cropping durations, differences in absorbance profiles reveal temporal changes in DOM composition under prolonged monocropping: specifically, the 6-year (6 y) and 12-year (12 y) soils showed absorbance shifts, likely reflecting the breakdown or alteration of DOM components (e.g., humic substances). These changes can be attributed to microbial degradation and modified organic matter inputs over cropping periods, which affect the aromaticity and stability of DOM in soil leachate [58]. This aligns with previous studies showing that prolonged monocropping reduces concentrations of certain DOM fractions due to depleted organic inputs and declined microbial activity [59,60], as evidenced by the observed loss of aromatic compounds and reduced DOM complexity over time.

#### 4.2.2. Role of DOM Fluorescence Characteristics in Soil Leachates

The highest SUVA_254_, SUVA_300_, and SUVA_350_ values in the 2 y soil indicate a higher aromatic compound concentration and DOM aromaticity/complexity compared to the 12 y soil (Figure 5). Their consistent decrease with increasing continuous cropping duration suggests depletion of stable, high-molecular-weight aromatic structures in soil organic matter, which aligns with shifts in microbial activity and organic matter decomposition [61]. The 2 y soil exhibited the highest E_2_/E_3_ ratio (indicating more low-molecular-weight, labile, and bioavailable DOM) and E_4_/E_6_ ratio (reflecting less humified organic matter). Both ratios declined with prolonged cropping, indicating accumulation of high-molecular-weight, highly humified DOM—consistent with microbial decomposition promoting stable organic matter that is less accessible for mineralization and nutrient cycling [62].

Additionally, the increasing SR (slope ratio) suggests a shift toward more terrestrial DOM inputs with long-term continuous cropping, likely driven by altered organic matter inputs and enhanced microbial degradation [63]. This transition reflects a shift from labile, easily decomposable DOM to recalcitrant, humified forms.

### 4.3. Effects of Continuous Cropping on the 3D Fluorescence Spectra of Water-Soluble

#### 4.3.1. Effects on Fluorescence Components

Fluorescence region distribution is widely used to characterize DOM components (e.g., protein-like and humic-like substances) [64]. Regions I and II (typical of protein-like materials) dominated the 2-year (2 y) soil, indicating that DOM was primarily derived from fresh plant/microbial residues—biologically active, labile, and less stable than humic substances [65]. With increasing continuous cropping duration, fluorescence spectra shifted: 6-year cropping promoted humic-like substance accumulation (more stable and less biologically active) [66]. reflecting gradual plant residue decomposition and formation of microbially recalcitrant complex organic molecules. In the 12-year (12 y) soil, further spectral changes indicated dominance of high-molecular-weight, stable humic-like substances, likely due to depletion of labile organic matter and accumulation of humified compounds [67]. Overall, continuous cropping significantly altered rhizosphere DOM composition, driving a shift from protein-like to humic-like substances. This transition may reduce labile organic matter availability, affecting soil fertility and microbial activity—consistent with previous studies on continuous cropping impacts on DOM quality and composition and their implications for soil nutrient cycling and microbial dynamics [68,69].

#### 4.3.2. Spectral Parameter Changes in DOM

The fluorescence indices (FI), humification index (HIX), and biological index (BIX) of dissolved organic matter (DOM) in the soil leachate were evaluated under different continuous cropping durations (2 y, 6 y, and 12 y). These parameters reflect key aspects of DOM composition, including aromaticity, degree of humification, and microbial origin, all of which are essential for understanding the quality and stability of organic matter in the soil [70].

As shown in Table 3, the shifts in spectral parameters reveal a significant transformation in DOM composition and origin driven by continuous cropping duration. The significant decrease in FI from 2 y to 12 y suggests that long-term continuous cropping results in a shift towards more humic, less biologically active material [71]. This is consistent with previous studies that have shown a decrease in protein-like material as the soil becomes more humified over time due to microbial decomposition and the accumulation of humic substances [72].

The concomitant decrease in HIX further suggests that long-term cropping leads to a higher degree of humification, accompanied by a reduction in the availability of labile, easily degradable organic matter. This trend supports the notion that continuous cropping enhances the stability of DOM by promoting humification and the accumulation of more stable organic compounds [73].

The higher BIX value in the 6 y and 2 y soils suggests that microbial processes play a more significant role in the formation of DOM in the early years of continuous cropping. In contrast, the lower BIX value in the 12 y soil indicates a shift towards terrestrial, humic sources of DOM, likely due to the reduced microbial activity and decomposition of plant residues over time [74].

Overall, the results from the spectral parameters indicate that continuous cropping significantly alters the composition and origin of DOM in the rhizosphere. The decrease in FI and BIX, combined with the lower HIX, suggests that long-term cropping may deplete easily degradable, biologically active organic matter, leading to an increase in more stable, humified substances. These changes could have important implications for soil fertility and microbial activity, as the availability of labile carbon sources decreases with prolonged cropping [75].

#### 4.3.3. Distribution of Fluorescent Components in Soil DOM

Shifts in fluorescent component distribution clarify the transformation of DOM quality induced by continuous cropping. The high abundance of protein-like components in the 2 y soil indicates that DOM is dominated by fresh, biologically active substances, reflecting intense microbial activity and recent organic inputs in the early stage of continuous cropping [76].

From 2 y to 12 y, the relative contribution of protein-like components decreased gradually, while humic-like (M) and terrestrial humic-like (T) substances increased simultaneously, showing a distinct transition in DOM composition. This indicates that prolonged continuous cropping promotes microbial degradation and humification of organic matter, leading to the accumulation of more stable and recalcitrant humic compounds [77]. In the 12 y soil, humified substances dominated with reduced labile protein-like materials, meaning long-term continuous cropping results in a DOM pool with enhanced stability but decreased biological activity, which is consistent with the depletion of labile carbon sources and reduced overall microbial activity under long-term monocropping [78].

Overall, data in Figure 7 show that continuous cropping significantly alters DOM composition by reducing the proportion of protein-like and biologically active compounds and increasing that of humic-like and terrestrial humic-like substances, reflecting the long-term effect of monocropping on soil organic matter (i.e., depletion of readily available organic materials and accumulation of stable humified organic matter), which may affect soil microbial activity and nutrient cycling [79].

### 4.4. Soil Microbial Bacterial Diversity

#### 4.4.1. Bacterial Community Structure

Continuous cropping across different time scales significantly altered soil bacterial community structure, with shifts in composition indicating ecological succession driven by changing soil environmental conditions. Copiotrophic Proteobacteria dominated the 2-year (2 y) soil—corresponding to a nutrient-rich environment supported by abundant root exudates and labile organic matter—before declining to 38% at 12 years (12 y), likely due to reduced labile carbon availability and slowed organic matter mineralization [80]. Concurrently, the oligotrophic phylum Acidobacteria increased from 15% to 22%, reflecting community adaptation to diminishing carbon resources and potentially more acidic conditions associated with complex organic compound accumulation [81,82]. Actinobacteria and Bacteroidetes declined from 10% to 7% over time, likely due to their reliance on labile carbon sources (e.g., root exudates) that became less available [83,84]. while Chloroflexi increased from 10% to 13% at 12 y—suggesting enhanced colonization by taxa involved in recalcitrant organic matter degradation and adaptation to oligotrophic/low-oxygen environments, linked to long-term organic matter humification and carbon stabilization [85].

At the genus level, *Nitrospira* (a key taxon in nitrogen cycling) decreased in relative abundance by 12 y, potentially associated with altered soil nitrogen availability [86]. Whereas *Sphingomonas*, MND1, and Gemmatimonadetes were more abundant in 12 y samples—attributed to their ability to degrade complex organics, adapt to low-oxygen/oligotrophic conditions, and cope with nutrient-poor soils [87,88]. Overall, bacterial communities underwent ecological succession: copiotrophic taxa dominated initially under high-nutrient conditions, while oligotrophic groups gradually prevailed as organic matter mineralization slowed and soils became more oligotrophic [89]. The rise in Chloroflexi and other specialist genera at 12 y reflects a shift from rapid organic matter decomposition to slower turnover, highlighting critical insights into long-term soil health under continuous cropping [90].

#### 4.4.2. Bacterial Community Diversity

Soil microbial diversity was assessed using several alpha diversity indices, including OTU, ACE, Chao1, Simpson, and Shannon indices (Table 4). The initial elevation in OTU, ACE, and Chao1 indices in the 2-year soil suggests a peak in microbial richness during the early stage of continuous cropping, likely fueled by the influx of readily decomposable organic substrates from fresh plant residues and root exudates. The subsequent stabilization of these richness indices, coupled with the absence of significant differences between the 6-year and 12-year groups, indicates that a new equilibrium in microbial richness is established after the initial cropping period.

The remarkable consistency in the Simpson index and the minimal fluctuation of the Shannon index around a high value (≥6.327) demonstrate that the evenness and overall diversity of the microbial community were largely preserved despite the progression of continuous cropping. These findings highlight that while continuous cropping may lead to a slight reduction in microbial richness from its initial peak, the structural diversity and equitability of the soil microbial community exhibit considerable resilience, stabilizing after a certain period of agricultural management [91].

### 4.5. Correlation Analysis Between Soil Nutrients, Bacterial Community Indices, DOM UV Absorption Indices, and 3D Fluorescence Spectroscopy Indices Under Continuous Cropping Conditions

The shifting correlation patterns from continuous cropping at 2 years (2 y) to 12 years (12 y) reveal a fundamental transition in the factors influencing microbial community dynamics under prolonged continuous cropping. In the initial 2-year stage (Figure 9a), the positive correlations of total organic carbon (TOC) and dissolved organic carbon (DOC) with diversity indices highlight the predominant role of labile carbon pools in supporting microbial richness and evenness. This suggests that in the early stages of cropping, readily available organic carbon from root exudates and fresh plant residues plays a crucial role in sustaining microbial diversity.

The concurrent negative correlation between SUVA254 (a UV-absorption index related to aromaticity) and richness indices (ACE, Chao1) provides early evidence that the increasing aromaticity of organic matter begins to constrain microbial community structure. As organic matter becomes more aromatic, it may become less bioavailable to microorganisms, limiting their ability to thrive in the soil environment [92].

By the 6-year stage (Figure 9b), the emergence of soil pH as a significant negative correlation with diversity indices suggests that progressive acidification becomes a critical factor reshaping microbial community structure. This change is likely driven by the inhibition of pH-sensitive bacterial taxa, as many microorganisms are sensitive to pH fluctuations, with lower pH potentially reducing the abundance of certain bacterial groups. The sustained positive influence of DOC, however, confirms the continued importance of available carbon substrates in sustaining microbial diversity [93].

In the 12-year soil samples, these trends were further reinforced, but the relationships between soil characteristics and microbial diversity became more complex (Figure 9c). For instance, SUVA254 showed significant negative correlations with both the Shannon and Simpson indices, suggesting that as soil humification increases, microbial diversity decreases. This decline in diversity could be attributed to the accumulation of humus and complex organic matter in the soil, which is less bioavailable to microbes compared to more labile organic material. Meanwhile, DOC continued to show positive correlations with the Shannon and Simpson indices, emphasizing the continued importance of available carbon sources in shaping microbial community structure [94].

Over the course of 2 to 12 years of continuous cropping, the correlations between soil physicochemical properties and microbial diversity indices underwent significant changes. As cropping duration increased, the level of humification in the soil organic matter gradually intensified, which in turn impacted on microbial community diversity and structure. The increase in DOC and TOC contributed to microbial community richness and diversity, indicating that organic carbon availability plays a central role in shaping microbial communities. However, the increase in soil acidity and humification appeared to suppress microbial diversity, indicating a shift in microbial community dynamics as the soil environment evolved over time [95].

### 4.6. Correlation Analysis of Soil Nutrients, Bacterial Diversity Indices, UV-Visible Spectral Indices, and 3D Fluorescence Spectral Indices

Partial Least Squares Path Modeling (PLS-PM) results clarify the complex interactions and temporal dynamics of soil ecosystems under continuous cropping (Figure 10). A strong negative path from cropping duration to soil physicochemical properties (R^2^ = 0.93, *p* < 0.001) indicates systematic soil health degradation, including depletion of organic matter and nutrient pools, which impairs nutrient retention and physicochemical stability, thereby affecting soil health and bacterial community dynamics [96].

Bacterial community indices (OTU, ACE, Shannon, Simpson) showed complex correlations with cropping duration—initial increases in microbial diversity may relate to early-stage nutrient availability—and significant associations with UV-visible/3D fluorescence spectral indices, as well as dissolved organic carbon (DOC) and total organic carbon (TOC), highlighting the key role of organic carbon in shaping microbial richness and evenness [97].

Specifically, UV-visible indices (e.g., SUVA_254_) correlated with microbial diversity (ACE, Chao1), reflecting the influence of organic matter humification on microbial community structure, while 3D fluorescence indices (FI, HIX, BIX) linked to microbial diversity underscored the role of organic matter decomposition in driving microbial complexity and ecosystem stability [98].

Overall, soil physicochemical properties, microbial diversity, and spectral indices are closely interconnected: early-stage increases in organic matter and microbial diversity are mirrored in spectral changes, while prolonged cropping leads to physicochemical deterioration and stabilized microbial diversity. UV-visible and 3D fluorescence indices effectively capture organic matter and microbial dynamics, revealing complex feedback mechanisms regulating soil health under long-term cropping. These findings suggest that monitoring soil nutrients and spectral indices can serve as effective tools for assessing microbial dynamics and cropping system sustainability [99].

## 5. Conclusions

This study systematically evaluated the effects of continuous cropping durations (2, 6, and 12 years) on soil physicochemical properties, dissolved organic matter (DOM) characteristics, and microbial communities associated with *Aralia continentalis* Kitag., with four predefined hypotheses fully validated.

Hypothesis 1 is supported: key soil physicochemical properties showed a significant declining trend with extended continuous cropping. Total and available nutrients (N, P, K) and total organic carbon (TOC) decreased progressively, while soil acidification was confirmed by a continuous pH reduction over the cropping period.

Hypothesis 2 is validated: the soil microbial community underwent a directional succession from copiotrophic to oligotrophic taxa. Copiotrophic phyla (e.g., Proteobacteria) decreased in relative abundance, while oligotrophic groups (e.g., Acidobacteria) increased and stabilized in the long term, accompanied by corresponding genus-level shifts.

Hypothesis 3 is confirmed: close linkages exist between soil DOM characteristics and microbial diversity. Dissolved organic carbon (DOC) and TOC showed robust positive correlations with microbial diversity indices. Concurrently, DOM composition transitioned dynamically from protein-like to humic-like fractions, synchronized with microbial community succession.

Hypothesis 4 is verified: UV-visible spectral indices (e.g., SUVA_254_, E_2_/E_3_, E_4_/E_6_) and 3D fluorescence spectral indices (e.g., FI, HIX, BIX) are effective proxies for assessing DOM quality and inferring microbial dynamics. These indices exhibited significant correlations with microbial diversity metrics, capturing critical shifts in DOM aromaticity, humification degree, and source that reflect underlying microbial community changes.

Regarding core findings, microbial alpha diversity increased from 2 to 6 years of cropping and then stabilized, with no significant differences between 6 and 12 years. DOM underwent a substantial transformation from labile, protein-like components to stable, humic-like fractions, driven by prolonged cropping practices and microbial decomposition. PLS-PM analysis confirmed that continuous cropping duration directly drove changes in soil physicochemical properties, which indirectly shaped microbial diversity and DOM characteristics, with organic carbon (DOC/TOC) acting as a key mediating variable.

This research establishes a holistic framework for understanding continuous cropping-induced soil degradation, highlighting the practical utility of spectroscopic techniques for non-destructive soil health monitoring. The insights gained provide valuable guidance for the sustainable cultivation of *A. continentalis* and analogous medicinal plants in temperate black soil regions, offering a foundation for evidence-based agricultural management practices.

## Figures and Tables

**Table 1:** Changes in UV fluorescence parameters of DOM under different continuous cropping durations (*n* = 3).

Group	SUVA_254_ (L·mg^−1^·m^−1^)	SUVA_260_ (L·mg^−1^·m^−1^)	SUVA_300_ (L·mg^−1^·m^−1^)	E_2_/E_3_	E_4_/E_6_	SR
2 y	0.8679 ± 0.0082 ^b^	0.8063 ± 0.0076 ^b^	0.5600 ± 0.0053 ^b^	3.2816 ± 0.0046 ^a^	17.860 ± 0.030 ^a^	0.850 ± 0.005 ^b^
6 y	1.2013 ± 0.0067 ^a^	1.1375 ± 0.0063 ^a^	0.7502 ± 0.0041 ^a^	3.5329 ± 0.0080 ^a^	17.676 ± 0.041 ^a^	1.004 ± 0.010 ^a^
12 y	0.8337 ± 0.0127 ^b^	0.7813 ± 0.0119 ^b^	0.5244 ± 0.0080 ^b^	3.4223 ± 0.0077 ^a^	16.960 ± 0.055 ^b^	0.931 ± 0.0080 ^a^

Note: Changes in UV-fluorescence parameters of dissolved organic matter (DOM) under different continuous cropping durations. SUVA_254_, SUVA_260_, and SUVA_300_ represent specific UV absorbance at 254 nm, 260 nm, and 300 nm, respectively (unit: L·mg^−1^·m^−1^), indicating the aromaticity and molecular weight of DOM. E_2_/E_3_ is the ratio of absorbance at 250 nm to 365 nm, E_4_/E_6_ is the ratio of absorbance at 465 nm to 665 nm, and SR is the spectral ratio, all reflecting the humification degree and molecular weight characteristics of DOM. Different lowercase letters within each column indicate significant differences between groups (*p* < 0.05).

**Table 2:** Fluorescence Characteristics of DOM under Different Continuous Cropping Durations (*n* = 3).

Group	Region I (Ex/Em 280/320 nm)	Region II (Ex/Em 300/420 nm)	Region III (Ex/Em 350/450 nm)	Region IV (Ex/Em 400/480 nm)	Region V (Ex/Em 420/500 nm)
2 y	1325 ± 30.2 ^a^	1159 ± 25.1 ^a^	663 ± 12.3 ^a^	497 ± 8.1 ^a^	331 ± 7.3 ^a^
6 y	2345 ± 50.4 ^b^	2050 ± 48.3 ^b^	1005 ± 14.5 ^b^	821 ± 12.2 ^b^	548 ± 10.9 ^b^
12 y	1916 ± 40.5 ^c^	1700 ± 37.9 ^c^	821 ± 18.3 ^c^	548 ± 15.2 ^c^	274 ± 8.3 ^c^

Note: Different letters within each column indicate significant differences between different groups (*p* < 0.05). The 3D-EEM spectra are divided into five regions based on the regional integration method: Region I (Ex < 250 nm, Em < 330 nm) and Region II (Ex < 250 nm, 330 nm < Em < 380 nm) represent protein-like substances (e.g., tyrosine and tryptophan derivatives); Region III (250 nm < Ex < 280 nm, Em < 380 nm) represents soluble microbial by-product-like substances; Region IV (280 nm < Ex < 350 nm, Em < 480 nm) and Region V (Ex > 350 nm, Em > 480 nm) represent humic-like substances (e.g., fulvic acid and humic acid derivatives). These regions reflect the compositional characteristics of DOM under different consecutive cropping durations.

**Table 3:** Influence of continuous cropping duration on fluorescence spectral parameters of DOM (*n* = 3).

Group	FI	HIX	BIX
2 y	1.7428 ± 0.0101 ^a^	0.7307 ± 0.0067 ^a^	0.6811 ± 0.0050 ^a^
6 y	1.7438 ± 0.0106 ^a^	0.6312 ± 0.0071 ^b^	0.7358 ± 0.0081 ^b^
12 y	1.6875 ± 0.0089 ^b^	0.6396 ± 0.0069 ^b^	0.6954 ± 0.0074 ^a^

Note: Influence of continuous cropping duration on fluorescence spectral parameters of dissolved organic matter (DOM). FI denotes Fluorescence Intensity, reflecting the total content of fluorescent substances in DOM. HIX represents Humification Index, characterizing the humification degree of DOM. BIX stands for Biological Index, indicating the contribution of biological sources to DOM. Different lowercase letters within each column indicate significant differences between groups (*p* < 0.05).

**Table 4:** Statistics of alpha diversity index of bacteria in samples (*n* = 3).

Group	OTU	ACE	Chao1	Simpson	Shannon
2 y	1673.33 ± 5.51 ^a^	1683.44 ± 5.16 ^b^	1686.90 ± 4.77 ^b^	0.0041 ± 0.0001 ^a^	6.376 ± 0.017 ^a^
6 y	1693.67 ± 4.51 ^a^	1699.82 ± 4.40 ^b^	1701.67 ± 4.42 ^a^	0.0042 ± 0.0001 ^a^	6.327 ± 0.017 ^a^
12 y	1696.00 ± 4.58 ^a^	1705.16 ± 4.29 ^a^	1706.46 ± 4.31 ^a^	0.0041 ± 0.0001 ^a^	6.376 ± 0.014 ^a^

Note: 1. Different lowercase letters within each column indicate significant differences between groups (*p* < 0.05), with “a” representing the highest value and “c” the lowest. 2. Abbreviations: OTU = Operational Taxonomic Unit (a unit used to classify microorganisms based on sequence similarity); ACE = Abundance-based Coverage Estimator (an index estimating microbial community richness, accounting for rare taxa not fully detected). 3. The Simpson index ranges from 0 to 1: values closer to 0 indicate higher microbial community diversity; values closer to 1 indicate lower diversity. The low Simpson index (~0.004) in this study reflects high bacterial diversity.

## Data Availability

The original contributions presented in this study are included in the article/Supplementary Material (Table S1). Further inquiries can be directed to the corresponding author.

## Supplementary Materials

The following supporting information can be downloaded at: https://www.mdpi.com/article/10.3390/biology14121750/s1, Table S1: Summary of the physicochemical properties of soil under different cropping durations (2 years, 6 years, 12 years).

## Abbreviations

The following abbreviations are used in this manuscript:

DOM	Dissolved Organic Matter
UV	ultraviolet
pH	potential of Hydrogen
SWC	Soil Water Content
TN	Total Nitrogen
TP	Total Phosphorus
TK	Total Potassium
AN	Available Nitrogen
AP	Available Phosphorus
AK	Available Potassium
DOC	Dissolved Organic Carbon
TOC	Total Organic Carbon
SUVA_254_	Specific Ultraviolet Absorbance at 254 nm
SUVA_260_	Specific Ultraviolet Absorbance at 260 nm
SUVA_300_	Specific Ultraviolet Absorbance at 300 nm
SR	Slope Ratio
FI	Fluorescence Indices
HIX	Humification Index
BIX	Biological Index
A	Excitation humic-like
C	protein-like
M	humic-like
T	terrestrial humic-like
B	biological humic-like
SEM	structural equation model
3D-EEMs	three-dimensional fluorescence spectra
Ex	excitation wavelength
Em	emission wavelength
OTU	Operational Taxonomic Unit
ACE	Abundance-based Coverage Estimator
