# Supplementary material for: Shifts in Dissolved Organic Matter and Microbial Communities Under Continuous Cropping of Aralia continentalis Kitag.: A Comparative Study of 2-, 6-, and 12-Year Durations
Source: Biology (Basel). 2025 Dec 6;14(12):1750. doi: 10.3390/biology14121750 (PMC12730357; doi:10.3390/biology14121750)
Supplement: Supplementary file 1 [file biology-14-01750-s001.zip › biology-3981945-supplementary.pdf]

Table S1: Soil Physicochemical Properties of *Aralia continentalis* Kitag. in Different Sampling Plots

| **sample plot** | **2y** | | | **6y** | | | **12y** | | |
|---|---|---|---|---|---|---|---|---|---|
| Repeat | **Plot 2 - Rep1** | **Plot 2 - Rep2** | **Plot 2 - Rep3** | **Plot 6 - Rep1** | **Plot 6 - Rep2** | **Plot 6 - Rep3** | **Plot 12 - Rep1** | **Plot 12 - Rep2** | **Plot 12 - Rep3** |
| **Soil Water Content (%)** | 20.35 | 18.89 | 19.24 | 25.22 | 24.98 | 26.31 | 22.18 | 22.64 | 21.97 |
| **pH** | 6.8 | 6.5 | 6.4 | 6.4 | 6.5 | 6.0 | 5.8 | 6.2 | 5.9 |
| **Porosity (%)** | 45.20 | 46.23 | 45.73 | 55.28 | 56.37 | 56.19 | 50.39 | 52.07 | 50.63 |
| **Electrical Conductivity (S/cm)** | 155.32 | 158.24 | 157.64 | 232.34 | 233.62 | 235.19 | 333.49 | 319.05 | 321.44 |
| **Total Organic Carbon (g/kg)** | 12.48 | 13.34 | 13.26 | 16.61 | 16.73 | 17.27 | 16.27 | 15.92 | 16.21 |
| **Total Nitrogen (g/kg)** | 0.89 | 0.95 | 1.02 | 1.22 | 1.35 | 1.26 | 0.68 | 0.66 | 0.71 |
| **Total Phosphorus (g/kg)** | 0.46 | 0.52 | 0.48 | 0.44 | 0.42 | 0.39 | 0.33 | 0.36 | 0.37 |
| **Total Potassium (g/kg)** | 8.63 | 8.98 | 9.55 | 6.68 | 6.79 | 7.02 | 6.58 | 6.69 | 6.39 |
| **Available Nitrogen (mg/kg)** | 73.12 | 75.33 | 77.27 | 83.42 | 80.25 | 79.24 | 63.22 | 66.21 | 60.94 |
| **Available Phosphorus (mg/kg)** | 16.33 | 16.35 | 16.48 | 16.27 | 16.02 | 15.97 | 13.08 | 13.61 | 13.44 |
| **Available Potassium (mg/kg)** | 104.35 | 105.28 | 104.77 | 100.28 | 101.36 | 102.44 | 90.48 | 87.29 | 88.37 |
| | | | | | | | | | |
| **Dissolved Organic Carbon (g/kg)** | 0.34 | 0.35 | 0.34 | 0.67 | 0.68 | 0.68 | 0.64 | 0.60 | 0.62 |

Note: "Plot 2/6/12" represent three different field sampling plots; "Rep1/Rep2/Rep3" represent three biological replicates for each plot.
